# Biomechanical forces enhance directed migration and activation of bone marrow-derived dendritic cells

**DOI:** 10.1038/s41598-021-91117-2

**Published:** 2021-06-08

**Authors:** Ji-Hun Kang, Hyun Joo Lee, Ok-Hyeon Kim, Yong Ju Yun, Young-Jin Seo, Hyun Jung Lee

**Affiliations:** 1 Department of Life Science, Chung-Ang University, Seoul, 06974 Republic of Korea; 2 Graduate School of Energy and Environment (KU-KIST Green School), Korea University, Seoul, 02841 Republic of Korea; 3 Department of Anatomy and Cell Biology, College of Medicine, Chung-Ang University, Seoul, 06974 Republic of Korea; 4 Department of Global Innovative Drugs, Graduate School of Chung-Ang University, Seoul, 06974 Republic of Korea

**Keywords:** Biological techniques, Biotechnology, Computational biology and bioinformatics, Immunology

## Abstract

Mechanical forces are pervasive in the inflammatory site where dendritic cells (DCs) are activated to migrate into draining lymph nodes. For example, fluid shear stress modulates the movement patterns of DCs, including directness and forward migration indices (FMIs), without chemokine effects. However, little is known about the effects of biomechanical forces on the activation of DCs. Accordingly, here we fabricated a microfluidics system to assess how biomechanical forces affect the migration and activity of DCs during inflammation. Based on the structure of edema, we proposed and experimentally analyzed a novel concept for a microchip model that mimicked such vascular architecture. The intensity of shear stress generated in our engineered chip was found as 0.2–0.6 dyne/cm^2^ by computational simulation; this value corresponded to inflammation in tissues. In this platform, the directness and FMIs of DCs were significantly increased, whereas the migration velocity of DCs was not altered by shear stress, indicating that mechanical stimuli influenced DC migration. Moreover, DCs with shear stress showed increased expression of the DC activation markers MHC class I and CD86 compared with DCs under static conditions. Taken together, these data suggest that the biomechanical forces are important to regulate the migration and activity of DCs.

## Introduction

Dendritic cells (DCs) are highly efficient antigen-presenting cells (APCs) that bridge innate and adaptive immunity. DCs are differentiated from bone marrow precursors and monocytes to reside within peripheral tissues as immature DCs^1–3^. Following the recognition of pathogenic antigens by DCs, antigens are internalized to be fragmented in the cytosol and are presented in the context of major histocompatibility complex (MHC) molecules on the cell surface. Simultaneously, DCs undergo maturation to increase mobility for migration and the expression of MHC/costimulatory molecules^4, 5^. Danger signals such as pathogen-associated molecular patterns (PAMPs) are critical for initiating the maturation process in DCs. Pattern recognition receptors, including toll-like receptors (TLRs), on DCs recognize PAMPs to trigger the activation of transcription factors such as a nuclear factor of activated T cells and nuclear factor-κB^6–9^. Mature DCs migrate fast to reach draining lymph nodes; this process is facilitated by a concentration gradient of the chemokines C–C motif chemokine ligand (CCL) 19 and CCL21 and sensed through the chemokine receptor C–C motif chemokine receptor 7, which is highly expressed on the surface of mature DCs^10^. Then, T cells recognize antigens in the context of MHC molecules on DCs and simultaneously interact with costimulatory molecules such as CD86 expressed on DCs^11, 12^. Consequently, naïve T cells are differentiated into effector T cells to initiate the adaptive immune response. You cannot alter accepted Supplementary Information files except for critical changes to scientific content. If you do resupply any files, please also provide a brief (but complete) list of changes. If these are not considered scientific changes, any altered Supplementary files will not be used, only the originally accepted version will be published.We didn't change any data which was accepted by reviewers and editor. Please use the same one that was submitted before. 

Importantly, although many researchers have studied the effects of biochemical signaling such as cytokine or chemokine signaling, on the key aspects of DC biology, the roles of biomechanical forces in DC regulation have not been fully elucidated. Because DCs are recruited into many abnormal sites or pathological microenvironments such as those associated with inflammation^13^, cancer^14^, or cardiovascular disorders^15^, DC function may be regulated not only by biochemical factors but also by biomechanical stimuli such as substrate stiffness or altered fluid flow in blood and lymphatic organs. For example, the phenotype and function of DCs are altered by the stiffness-controlled two dimensional polyacrylamide gel substrate^16^ or collagen based 2D and 3D matrices^17^, suggesting that biomechanical forces may modulate the phenotype and function of DCs under pathological conditions. Moreover, several clinical studies have demonstrated the involvement of DCs in vascular pathology^15, 18^. Indeed, vascular sites in atherosclerosis reveal high wall strain by shear stress and/or enhanced stiffness^19^ and exhibit elevated numbers of DCs^18^. In healthy arterial walls, vascular DCs are found in an immature state, and the mature DC phenotype is prevalent during the onset of atherosclerosis^20^. Although atherosclerotic plaques produce the inflammatory cytokines recruiting DCs, altered hemodynamic flow by plaque formation in the vessels might be able to affect DC functions. Thus, we hypothesize that DC migration may be affected by altered biomechanical forces, such as interstitial pressure or fluid induced by edema at sites of inflammation.

In this study, we fabricated a novel microfluidic channel mimicking inflammatory edema using soft lithography. Our results showed that biomechanical force influenced the directed migration and activation of DCs. Importantly, the DC migratory pattern was effectively promoted, and DC activation was also increased in the shear stress environment, although migrating velocity was not changed. Thus, our study suggested that the shear stress generated by an interstitial fluid-like situation may stimulate DCs.

## Results

### Fabrication and characterization of microfluidic channels

**Figure 1:**
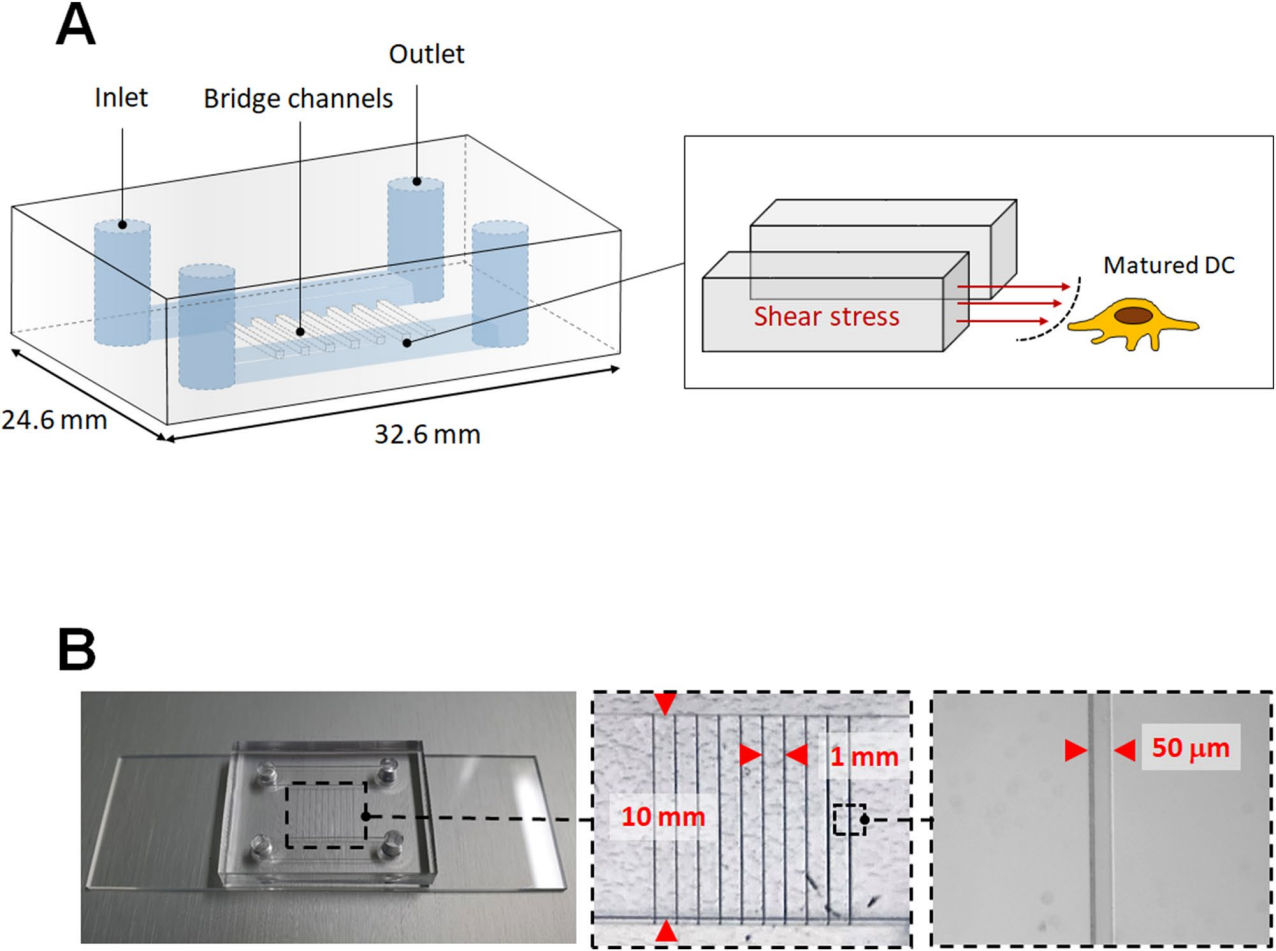
Fabrication and characterization of the microfluidics channel. (**A**) Schematic showing the layout of the microfluidics channel. Transparent view showing two main channels and bridge channels between the main channels. Cartoon showing how DCs were exposed to shear stress from the bridge channels. (**B**) Actual photograph of the microfluidic channel. Dimensions of the bridge channels are depicted with magnified images.

Tissue swelling, also known as edema, is one of the hallmarks of inflammation, and DCs are found in interstitial fluid related to inflammation. Mature DCs are sensitive to barotaxis generated from surrounding fluid and exhibit biased movement in bifurcated channels^21^. Therefore, we hypothesized that inflammatory fluid or interstitial fluid could affect DC migration towards lymphatic drainage and DC activation. To mimic interstitial fluid in this study, we developed an edema model consisting of two main channels connected by multiple bridge channels (Fig. 1A). Interstitial flow is the slow movement of fluid around cells and through the pores of the extracellular matrix, which comprises the interstitium. Shear stresses of interstitial flow are estimated to be less than 0.2–1.0 dyne/cm^2^^22^, and the velocities of interstitial flow are believed to range from 0.1 to 1.0 μm/s^23^. Murine BMDCs (1 × 10^6^) were resuspended in medium containing 1 μg/mL lipopolysaccharide (LPS) and seeded on type I collagen coated surface in the lower channel. Although type I collagen helps to define the functional properties of tissues^19^ and enables to provoke phenotypic changes associated with activation has been described in dendritic cells^24^, low concentration of collagen (50 µg/ml) was used for minimizing the effect of extracellular matrix on DC function in this study. After 24 h to permit cell adaptation, a programmable peristaltic pump was used to manage the laminar flow of medium through the upper channel at a constant flow rate. Immediately after the pump was started, the medium entered through the bridge channel and crossed to the lower channel, where DCs were plated, and mature DCs were then exposed to interstitial fluid, resulting in shear stress (Fig. 1A). Figure 1B shows the as-prepared microfluidic chip designed to mimic conditions of interstitial fluid related to inflammation. We constructed hybrid channels with a combination of a microscale square channel and millimeter-scale rectangular channels on a single chip. The center of the chip consisted of ten microchannels with a width of 50 μm, height of 50 μm, and length of 10 mm.

### Computational simulation of interstitial fluid-like shear stress

**Figure 2:**
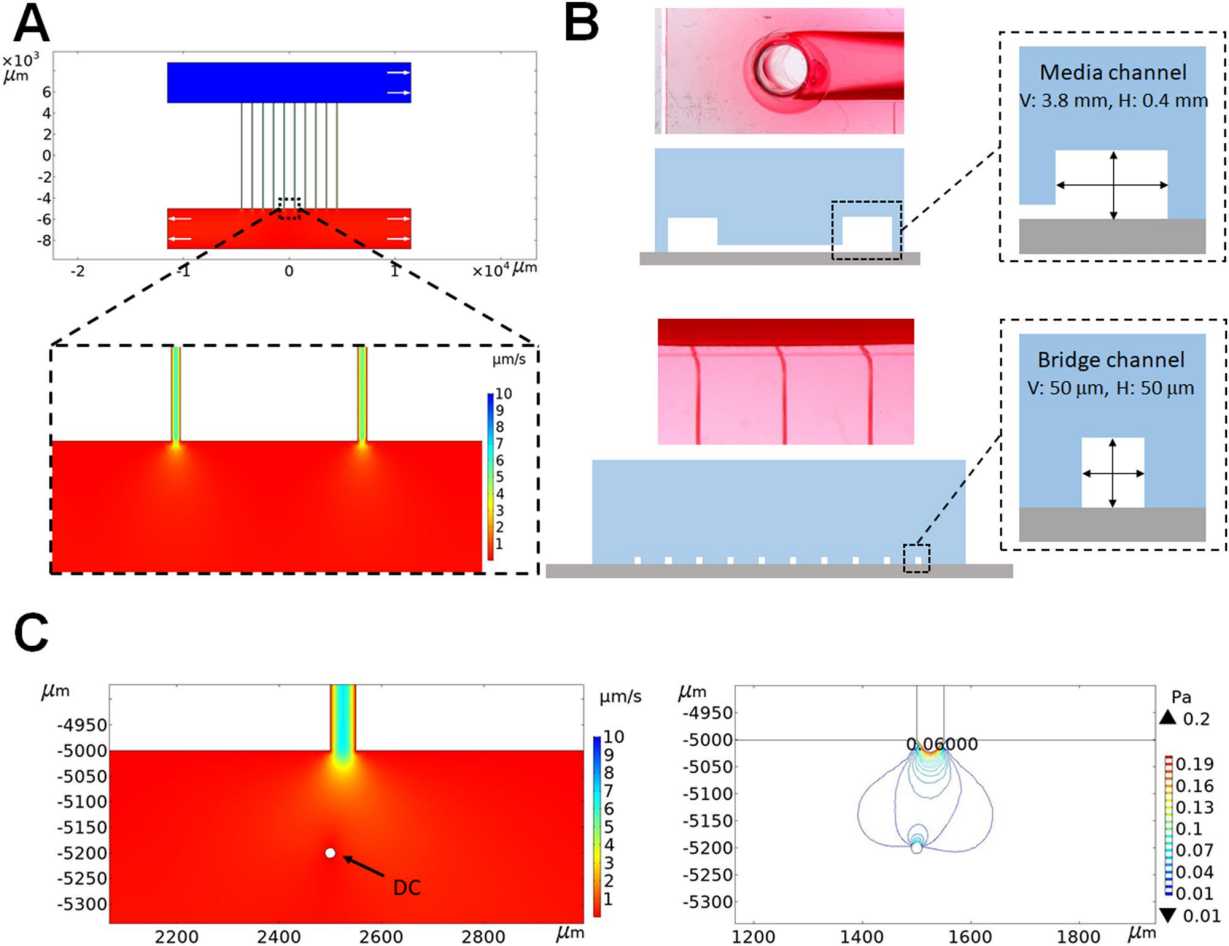
Computational modeling of inflammation mimicked by the microchannels. (**A**) COMSOL-based fluid velocity profile in the cell culture chamber (COMSOL Multiphysics 5.5 software, https://www.comsol.kr/release/5.5). The color scale represents the magnitude of velocity at any point under steady state conditions. White arrows represent the flow direction. (**B**) Optical image and cross-sectional schematic of the microfluidics chip. (**C**) Flow velocity and shear stress profiles of DCs.

To understand the fluid shear stress patterns experienced by the DCs cultured within the microchannels of our newly designed microfluidics system, we performed COMSOL-based computational simulations using two-dimensional solutions from the Laplace equation. The detailed modeling information is summarized in the supporting information. Figure 2A shows the COMSOL simulation results of the flow state of the fluid in the engineered microfluidic chip. In this design, 10 microscale channels were a source of media input into the cell culture area (Fig. 2B). Therefore, the flow velocity in the cell culture area largely decreased in the range of 0.1 to 2.0 μm/s. We also estimated the flow velocity and shear stress experienced by DCs. For DCs near microchannels, the maximum flow velocity and maximum shear stress exerted on the DCs were ~ 2.0 μm/s and ~ 0.6 dyne/cm^2^, respectively. In our system, the variation in shear stress was between 0.2 and 0.6 dyne/cm^2^. Based on two computational outputs, we determined that the cell culture area resulted in a uniformly distributed flow velocity of 0.1–2.0 μm/s and shear stress of 0.2–0.6 dyne/cm^2^ (Fig. 2C).

### Shear stress potentiates the migrating ability of DCs

**Figure 3:**
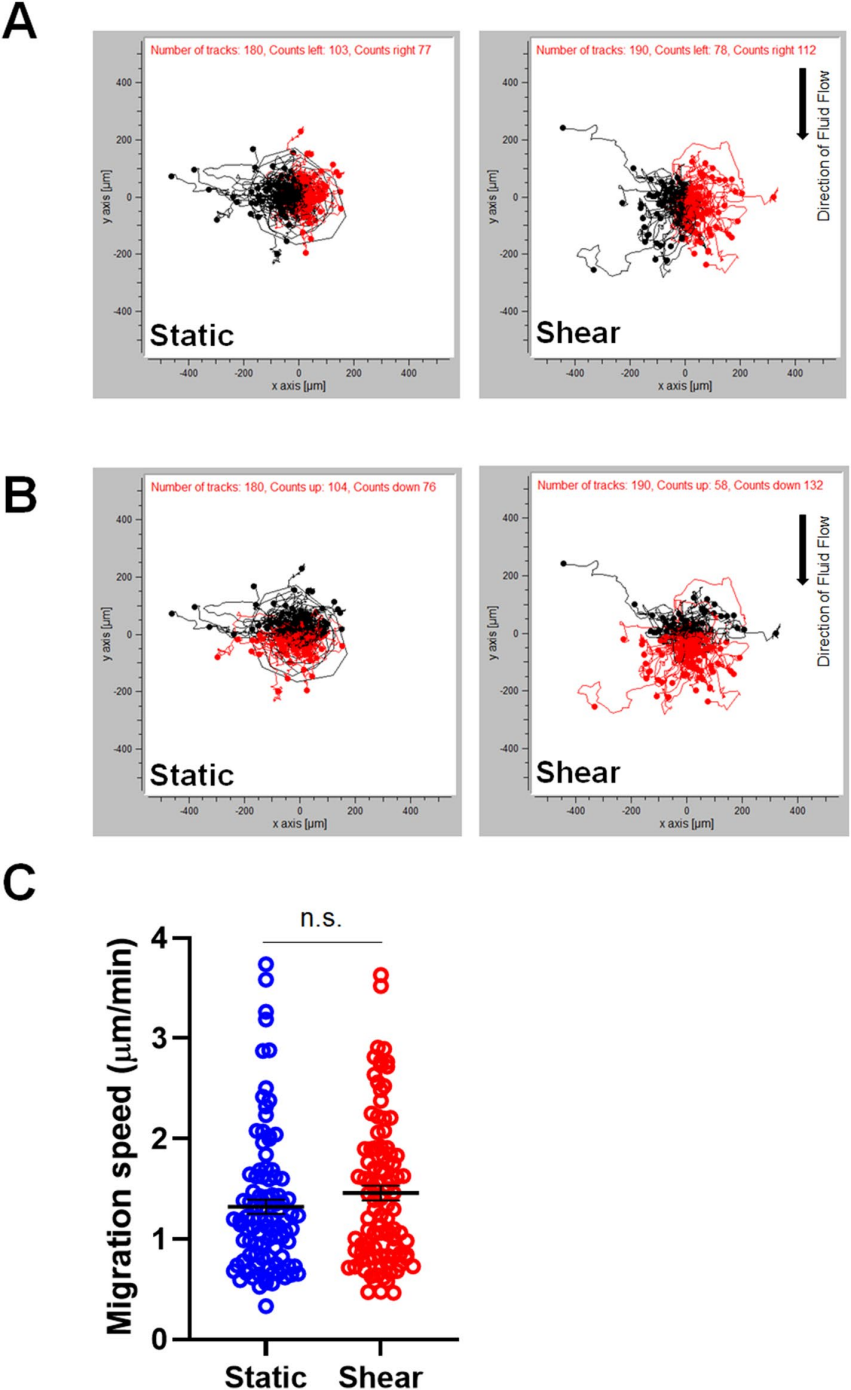
Shear stress does not alter the migration velocity of DCs. (**A**) and (**B**) Spatial tracking of DC movement during 5 h of time-lapse imaging where each cell lies at the origin (0,0) at *t* = 0 h. Plots depict the motility of individual cells in one representative experiment. Cells are marked as black/red dots for their movement toward left and right (**A**) and up and down (**B**). (**C**) Quantification of migration speed revealed the cellular velocities of individual cells following exposure to shear stress (image j, http://rsb.info.nih.gov/ij/; n = 3 independent experiments; unpaired *t*-test, *p* = 0.1775).

**Figure 4:**
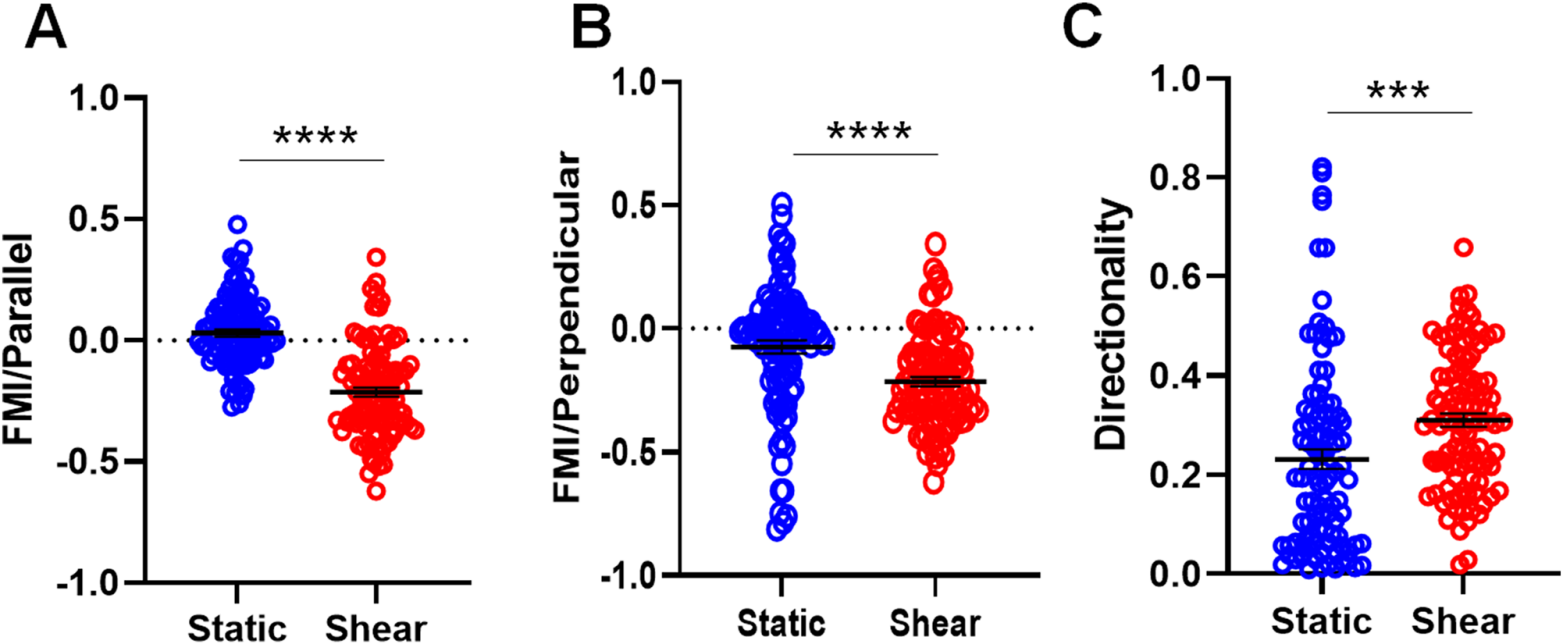
Shear stress affects the migratory patterns of DCs. Quantitative analysis of migratory behaviors according to (**A**) parallel FMI, (**B**) perpendicular FMI, and (**C**) directness of individual cells by shear stress (Chemotaxis and Migration tool, http://www.ibidi.com; n = 3 independent experiments; unpaired *t*-test, ***p ≤ 0.001, ****p ≤ 0.0001).

In order to investigate the effect of biomechanical force on DC migration, mouse BMDCs were seeded on type I collagen coated channel. When DCs were exposed to 0.2–0.6 dyne/cm^2^ shear stress, we monitored the migratory behaviors of DCs by analyzing time-lapse images from cultures exposed to shear stress or static conditions for 5 h. Analysis of migratory behaviors was performed for parameters testing speed and directionality (velocity, directness, and parallel and perpendicular FMIs) in these populations of migrating DCs with or without shear stress (“Methods” for detail). The overall cellular velocity of DCs was not altered by shear stress (1.323 ± 0.139 μm/min static versus 1.459 ± 0.145 μm/min shear stress; *p* = 0.1775; n = 3 independent experiments [means ± standard errors of the means]; Fig. 3A,C). Although migration speed of BMDCs was not altered by shear stress, BMDCs tend to follow the direction of fluid flow (Fig. 3B). In order to analyze the migrating pattern of BMDCs more detail, FMIs and directness were further assessed. Despite a lack of differences in cellular velocity between shear stress and static conditions, shear stress strongly attracted BMDCs, as indicated by the increase in the negative parallel and perpendicular FMI values relative to those under static conditions (Fig. 4A,B). Medium entered into the inlet located in the left corner of the upper channel and exited through the outlet in the right corner of the same channel. Thus, the medium in the lower channel also tended to move from left to right. The parallel FMI also showed a negative value in the shear stress group, indicating that DC migration may be followed by flow of fluid. In addition, directness indicated that migration of BMDCs followed rather straightforward trajectories and that these cells were significantly guided by shear stress (Fig. 4C). In contrast, static conditions resulted in nonspecific FMI and directness, indicating reduced guidance toward biomechanical forces.

### Shear stress increases the activation of DCs

**Figure 5:**
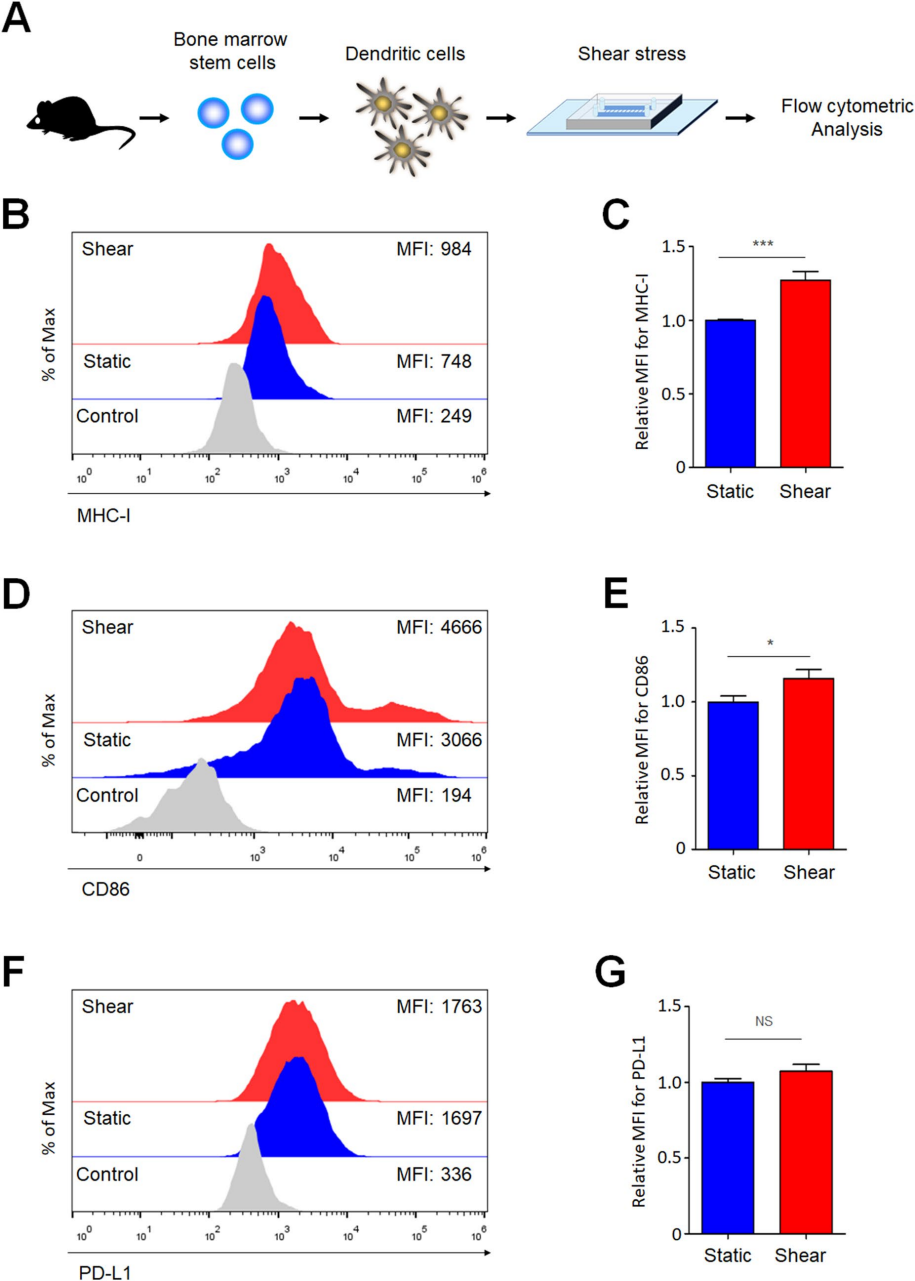
Shear stress increases the expression of MHC class I and CD86 on DCs. (**A**) BMDCs were seeded at a density of 10^6^ cells/mL in the microfluidic device in the presence or absence of 1 μg/mL LPS. After 24 h, BMDCs were either unexposed (static) or exposed to shear stress for 3, 6, or 12 h. Activation of BMDCs was analyzed by flow cytometry. (**B**,**C**) Representative histogram with geometric mean fluorescence intensity (MFI) (**B**) and relative mean fluorescence intensity (MFI) (**C**) of the MHC class I molecule are shown. (**D**,**E**) Representative histogram (**D**) and relative MFI (**E**) of CD86 are shown. (**F**,**G**) Representative histogram (**F**) and relative MFI (**G**) of PD-L1 are shown. The MFI of BMDCs under static conditions was set to 1.0. Data represent the means ± standard errors of the mean. *p ≤ 0.01; ***p ≤ 0.001; NS, not significant. CTR, LPS-untreated control. Representative data from at least three independent experiments are shown.

Migration ability is closely associated with the activation of DCs during inflammation^25^. Since we observed that shear stress changed the migration patterns of DCs (Fig. 4), and the activation of DCs may be influenced by shear stress as well. To test this hypothesis, BMDCs were exposed to shear stress to measure their activation by flow cytometry (Fig. 5A). Because DCs present antigens in the context of MHC molecules for T cells, higher expression of MHC molecules can induce stronger T-cell responses^26–28^. As previously known^29, 30^, LPS treatment increased expression levels of MHC-I (Fig. 5B). Interestingly, shear stress-exposed BMDCs expressed significantly higher levels of MHC class I molecules compared with BMDCs under static conditions (Fig. 5B,C). DCs express several costimulatory molecules including CD83, CD80, and CD86. CD83 is a member of the immunoglobulin (Ig) superfamily and is most highly and stably expressed by mature dendritic cells to regulate maturation and activation^31^. CD80 (B7-1) and CD86 (B7-2) are well known as the B7 family of costimulatory molecules and both bind to CD28 on the surface of naïve T cells. Although all these proteins are important for T cell stimulation, CD86 has been reported to be more important to generate adaptive immune responses than others. Therefore, we analyzed the expression level of CD86 as a representative costimulatory molecule of the activated DCs^32^. Similar to MHC-I, shear stress increased the expression of the costimulatory molecule CD86 on BMDCs (Fig. 5D,E). Although PD-L1 expression increases in some tumor cells upon shear stress^33^, we did not observe a significant increase in PD-L1 on shear stress-exposed BMDCs (Fig. 5F,G). Collectively, these results indicate that shear stress induces the activation of DCs.

## Discussion

DCs are important cells for induction of cellular and humoral immunity. Moreover, activation of natural killer (NK) cells and NK T cells is regulated by DCs^34^. Thus, many clinical trials are going on to assess immunotherapy for cancer and immune disorders^35, 36^. The biological properties of DCs in response to biomechanical stimuli in the extracellular space during inflammation still need to be elucidated. As DCs are activated during inflammation, when the amount of interstitial fluid is increased, we hypothesized that shear stress at inflammatory sites may also be an important factor enhancing the migration ability of DCs. To test this hypothesis, we developed a novel microfluidic channel system that mimicked conditions for interstitial fluid of inflammation. Interestingly, we found that shear stress increased the directed migration and activation of DCs. Diverse biochemical substances such as TLR4 (LPS) and TLR3 (Poly I:C) agonists are used to target antigen-presenting cells such as DCs resulting in increased potency and longevity of antigen-specific adaptive immune responses. However, the use of these molecules is often limited owing to the generation of unwanted nonspecific immune responses. Therefore, identification of the novel mechanisms of DC activation is essential for successful treatment of a variety of immune disorders, including infection, allergy, inflammation, and cancers.

Little is known about the effects of mechanical stimuli during inflammation on DC function. In this study, we generated microfluidics chip measuring 0.2–0.6 dyne/cm^2^ and cultured DCs within these chips, resulting in exposure to fluid shear stress, which may correspond to that encountered during inflammation or interstitial fluid flow. In our study, fluid shear stress enhanced the directness and FMI of DC migration. Directed migration of cells is essential in inflammation, immune system development, pathogen survival strategies, and allergic reactions^37^. Additionally, DC migration is determined by chemokines^37^, inflammatory cytokines^38^, growth factors^39^, modulating factors^40^, and integrins^41^. Moreover, our results suggested that the biomechanical forces induced by inflammation regulated DC migration and activity. Similarly, Xu et al.^42^ reported that the RhoGAP/RhoA/ROCK signaling axis regulates the directed migration of mature DCs to secondary lymphoid organs. In their study, RhoGAP-defective BMDCs showed less FMI and directness in response to the chemoattractant CCL21 compared with wild-type BMDCs on a two-dimensional fibronectin substrate. Interestingly, migration velocity was not changed in RhoGAP-defective BMDCs compared with that in wild-type BMDCs. Since RhoGAP also acts as a mechanosensor^43^, we postulated that shear stress-induced DC migration may also be related to the migration mechanisms mediated by the chemoattractant CCL21. Rho-GAP also regulates cytoskeletal rearrangements, which are important for establishing proper DC-T cell interactions between stimulated DCs and T cells in regional lymph nodes^42, 43^. Moreover, cytoskeletal rearrangement through F-actin dynamics could be modulated by biomechanical forces such as shear stress^43^. However, the specific pathways and factors controlling DC migration via biomechanical force need to be determined through future studies.

In this study, we used LPS to induce activation of DCs, followed by exposure to shear stress. Although LPS is a bacterial component, LPS is widely used to induce the expression of antigen presentation-associated proteins including MHC-I, MHC-II, and costimulatory molecules on DCs. Importantly, DCs as the most professional antigen-presenting cells can present exogenous antigens in the context of MHC-I through cross-presentation^44^, which enables DCs to generate CD8+ T cell responses. Therefore, enhanced expression of MHC-I on DCs stimulated with LPS could be important for the generation of cellular immune responses against intracellular bacteria and viruses without being infected. We observed that shear stress did not greatly increase the expression of activation markers including MHC-I and CD86 when compared to static condition. Since DCs were treated with LPS prior to exposure to shear stress, it is possible that expression of these activation markers was saturated, which results in subtle difference. However, this increase of activation markers in conjunction with enhanced migration ability might strongly impact DC's ability to induce T cell responses in vivo, which requires further investigation in the future study.

Dendritic cells are by far the most potent antigen-presenting cells owing to their unique antigen processing and presentation mechanisms. Thus, many preclinical and clinical studies have been performed to obtain an optimal DC vaccine or immunotherapy for treating immune disorders. To enhance DC activity and longevity, various chemokine ligands, cytokines, or even genetic modifications have been applied^45, 46^. Unfortunately, lack of efficacy is often observed when any single approach is applied, and effective therapies may require combinations of these approaches. Further studies are needed to improve the functionality or practicality of DC therapy. Thus, microfluidic platforms that mimic the DC microenvironment may be supporting tools for enhancing DC function.

## Methods

### Reagents and antibodies

For flow cytometric analyses, anti-mouse CD11c (N418) (Tonbo, San Diego, CA, USA), anti-mouse CD274 (PD-L1, B7-H1) (10F.9G2) (Tonbo), anti-mouse programmed death ligand 1 (PD-L1; Biolegend, San Diego, CA, USA), anti-mouse CD86 (GL-1) (Biolegend), and anti-mouse H-2Kb (Biolegend) antibodies were used according to the manufacturer’s instructions.

### Differentiation of mouse bone marrow-derived DC (BMDC)

Bone marrow cells were isolated from 6 to 7-week-old C57BL/6 male mice (DBL, Korea). Briefly, femurs and fibulas were separated from the hind legs of mice and cleared of muscle and tissue. Both ends of the bones were removed, and bone marrow was isolated from the bone barrel using a syringe containing phosphate-buffered saline (Welgene, Korea). Red blood cells were eliminated using ACK lysing buffer (Thermo). Bone marrow cells were resuspended in RPMI-1640 media (Welgene) supplemented with 10% fetal bovine serum (Welgene) and 1% penicillin/streptomycin (Welgene) in the presence of recombinant murine granulocyte–macrophage colony stimulating factor (Peprotech). Cells were then incubated at 37 °C in an atmosphere containing 5% CO_2_. Six days later, differentiation into DCs was evaluated on a flow cytometer. Experiments with mice were performed according to the protocol approved by the Institutional Animal Care and Use Committee at Chung-Ang University (Approval No. 201700089). All experiments were carried out in accordance with relevant guidelines and regulations. The study was carried out in compliance with the ARRIVE guidelines.

### Design and fabrication of two types of microchannel molds

The lymphatic microfluidic model was a two-layer polydimethylsiloxane (PDMS)-based chip fabricated using soft lithography. Two types of master molds on silicon wafers for the flow channel were produced by conventional photolithography^47^. First, a flow channel mold containing dozens of micro-scale channels was spin-coated with a SU-8 50 photoresist (Microchem, Newton, MA, USA) at 2000 rpm for 30 s to reach a final height of 50 μm. The photoresist was exposed to ultraviolet (350–400 nm) light using a contact Mask Aligner (Karl Suss MJB3) for 60 s to achieve a 50-μm thickness. After ultraviolet light (UV) exposure, postexposure bake was performed for 1 min at 65 °C and then 5 min at 95 °C. The master was then developed in an SU-8 developer (Microchem) to remove the uncrosslinked photoresist. Second, a flow channel mold with hundreds of micro-scale channels was spin-coated with SU-8 100 photoresist at 1000 rpm for 30 s to reach a final height of 300 μm and soft baked at 95 °C for 90 min. The photoresist was exposed to UV light for 60 s to achieve a 300-μm thickness. After UV exposure, postexposure bake was performed for 1 min at 65 °C and then 20 min at 95 °C. The master was then developed in an SU-8 developer for 20 min. Third, the prepared two flow molds were non-stick functionalized with trichlorosilane for 1 h in a vacuum desiccator. Fourth, PDMS precursor and curing agent were mixed at 10:1 by mass and cast on the prepared SU-8 masters to create channels for creating of the lymphatic microfluidic vessel. Finally, fully assembled chips were bonded to glass bottoms using oxygen plasma treatment.

### Application of shear stress

Before seeding cells in the devices, the channel was sterilized at 121 °C for 15 min and coated with 50 μg/mL type I collagen at 4 °C for 24 h. The next day, cells were directly seeded in the lower channel of the microfluidics channels at a final density of 10^6^ cells/mL. Following a 24-h culture period in the device, medium was injected into the upper channel where cells were not seeded and flushed at a constant flow rate γ using a programmable peristaltic pump (Longer Precision Pump, China). Shear rate created at the inner surface of the channel was calculated as γ = $\frac{({{m}}+2){{f}}}{{{\pi}}{{{\gamma}}}^{3}}$, and WSS (τ) as, τ = ηγ, where *f* is the total flow, γ is the internal radius of the channel, and η is the fluid viscosity. The values of the dimensionless number *m* depend on flow conditions. For laminar flow, *m* = 2, and for turbulent flow, *m* > 2^48^. We applied flow rates of 1440 μL/min, corresponding to values of 4 dyne/cm^2^ in the upper channel (Fig. 1A). The medium entered through the upper channel and the bridge channel, and cells in the lower channel (Fig. 1A) were exposed to the shear stress generated through the bridge channel. The mechanical forces experienced by the cells in the lower channel were calculated with the computational simulation described below.

### Computational simulation of fluid flow velocity and pressure in microfluidic channels

COMSOL Multiphysics 5.5 software was used to develop a finite element model of interstitial flow through the microchannels. The microchannel dimensions were imported from the AutoCAD file used to fabricate the lithography mask for the silicon master. The detailed modeling information is summarized in the supporting information. Channel layout and dimensions were taken from the device design. A Poiseulle boundary condition was applied at the inlet, with a zero-pressure outlet and a no-slip fluid-wall interface. This finite element simulation solved the Navier–Stokes equation to calculate the velocity profile inside and outside a rectangular bridge channel with a height of 50 μm and a width of 50 μm (Fig. 2A).

### Time-lapse imaging

For cell migration tracking experiments, PDMS channels were placed on an inverted microscope (Nikon Ti2), and cellular motility was observed with phase-contrast microscopy under static or shear stress conditions. At a given flow rate, successive images were recorded every 3 min for 5 h in an environmental chamber maintained at 37 °C with 5% CO_2_. Cells were tracked in time-lapse image sequences using the manual tracking plug-in included in the FIJI bundle for Image J 1.52a (http://rsb.info.nih.gov/ij). Image J output was integrated into Chemotaxis and Migration Tool software (ibidi GmbH, Germany) to determine cellular behavior in each channel. Total distances and average velocities were obtained from 180 to 190 cells for each group (> 60 cells per each experiment, 3 independent experiments by new batch of cells). For quantitative evaluation of directed cell migration, several parameters of the trajectories were measured, as described by Ruez et al.^49^. Here, we modified the parameters to provide indications regarding how fast and straight cells moved and how much this movement was directed toward the biomechanical forces. There were three parameters as follows. First, velocity represented absolute cell speed, whatever the direction, and was calculated by dividing the accumulated distance by the time length of the cell tracking for each cell. Values are expressed as μm/min and represent the average velocity of n cells for static and shear conditions $\frac{Track\;length}{Time}$.

Second, the forward migration indices (FMIs) parallel and perpendicular to the direction of flow represented the efficiency of forward migration of cells in relation to the axis parallel and perpendicular to the flow direction, respectively. Values represent the average FMIs parallel and perpendicular to n cells for static and shear conditions.

$$FMI/perpendicular = \frac{1}{n}\sum_{i=1}^{n}\frac{{x}_{i,end}}{{d}_{i,accum}}$$

$$FMI/parallel =\frac{1}{n}\sum_{i=1}^{n}\frac{{y}_{i,end}}{{d}_{i,accum}}$$

*Calculation of the FMI/perpendicular and the FMI/parallel: i* = *index of single cells, n* = *number of cells, x*_*i,end*_*, y*_*i,end*_ = *coordinates of the cells’ endpoints, d*_*i,accum*_ = *accumulated distance of the cells’ paths.*

Third, the directness represented a measure of how straight forward the cell trajectories were, whatever the direction. This parameter was calculated by dividing the Euclidian distance by the accumulated distance for each cell. Values represent the average directness of n cells for static and shear conditions.

$$Directness \; of \; single \;cells =\frac{{d}_{i,euclid}}{{d}_{i,accum}}$$

$$Averaged \; Directness = \frac{1}{n}\sum_{i=1}^{n}\frac{{d}_{i,euclid}}{{d}_{i,accum}}$$

*Calculation of the directness of cells: i = index of the single cell, d*_*i,Euclid*_* = Euclidean distance, d*_*i,accum*_* = accumulated distance.*

### Fluorescence-assisted cell sorting (FACS) analysis

BMDCs that were collected from PDMS channels were stained with antibodies on ice for 20 min. Cells were then washed with FACS buffer (1% bovine serum albumin, 10% sodium azide, and 2 mM ethylenediaminetetraacetic acid) twice and analyzed on an Attune NxT Acoustic Focusing Cytometer (Invitrogen, Carlsbad, CA, USA). Geometric mean fluorescence intensity (MFI) was determined for 10,000–30,000 live events.

### Statistical analysis

Prism (GraphPad) was used to analyze the significance. Student t-tests were used for comparisons between the two groups. Results with *p* values of less than or equal to 0.05 were considered statistically significant. All experiments were repeated at least three times, and each experiment was analyzed independently (Supplementary Information).Supplementary Information was received; however, no citation was provided in the manuscript. Please check and confirm the inserted citation. Otherwise, kindly advise us on how to proceed.We uploaded 'Supplementary Information' file through this system. Please find it. 

## Supplementary Information

Supplementary Information.
