# Supplementary material for: Biomechanical forces enhance directed migration and activation of bone marrow-derived dendritic cells
Source: Sci Rep. 2021 Jun 8;11:12106. doi: 10.1038/s41598-021-91117-2 (PMC8187447; doi:10.1038/s41598-021-91117-2)

SUPPLEMENTARY INFORMATION

Biomechanical forces enhance directed migration and activation of bone marrow-derived dendritic cells

**Ji-Hun Kang^1+^, Hyun Joo Lee^2+^, Ok-Hyeon Kim^3,4^, Yong Ju Yun^2*^, Young-Jin Seo^1*^, and Hyun Jung Lee^3,4*^**

^1^Department of Life Science, Chung-Ang University, Seoul 06974, Republic of Korea

^2^Graduate School of Energy and Environment (KU-KIST Green School), Korea University, Seoul 02841, Republic of Korea

^3^Department of Anatomy and Cell Biology, College of Medicine, Chung-Ang University, Seoul 06974, Republic of Korea

^4^Department of Global Innovative Drugs, Graduate School of Chung-Ang University, Seoul 06974, Republic of Korea

*corresponding authors

Yong Ju Yun, Ph.D.

Graduate School of Energy and Environment (KU-KIST Green School), Korea University, Seoul 02841, South Korea, Tel: +82-2-3290-5973, E-mail: [yjyun0@korea.ac.kr](mailto:yjyun0@korea.ac.kr)

Young-Jin Seo, Ph.D.

Department of Life Science, Chung-Ang University, 84 Heuksuk-ro, Dongjak-ku, Seoul 06974, South Korea, Tel: +82-2-820-5925, E-mail: [yjseo@cau.ac.kr](mailto:yjseo@cau.ac.kr)

Hyun Jung Lee, Ph.D.

Department of Anatomy and Cell Biology, College of Medicine, Chung-Ang University, 84 Heuksuk-ro, Dongjak-ku, Seoul 06974, South Korea, Tel: +82-2-820-5434, E-mail.: [pluto38@cau.ac.kr](mailto:pluto38@cau.ac.kr)

**Computational simulation of fluid flow velocity and shear stress in microfluidic channels using COMSOL software (5.5 version).**

- 1. Geometry 1


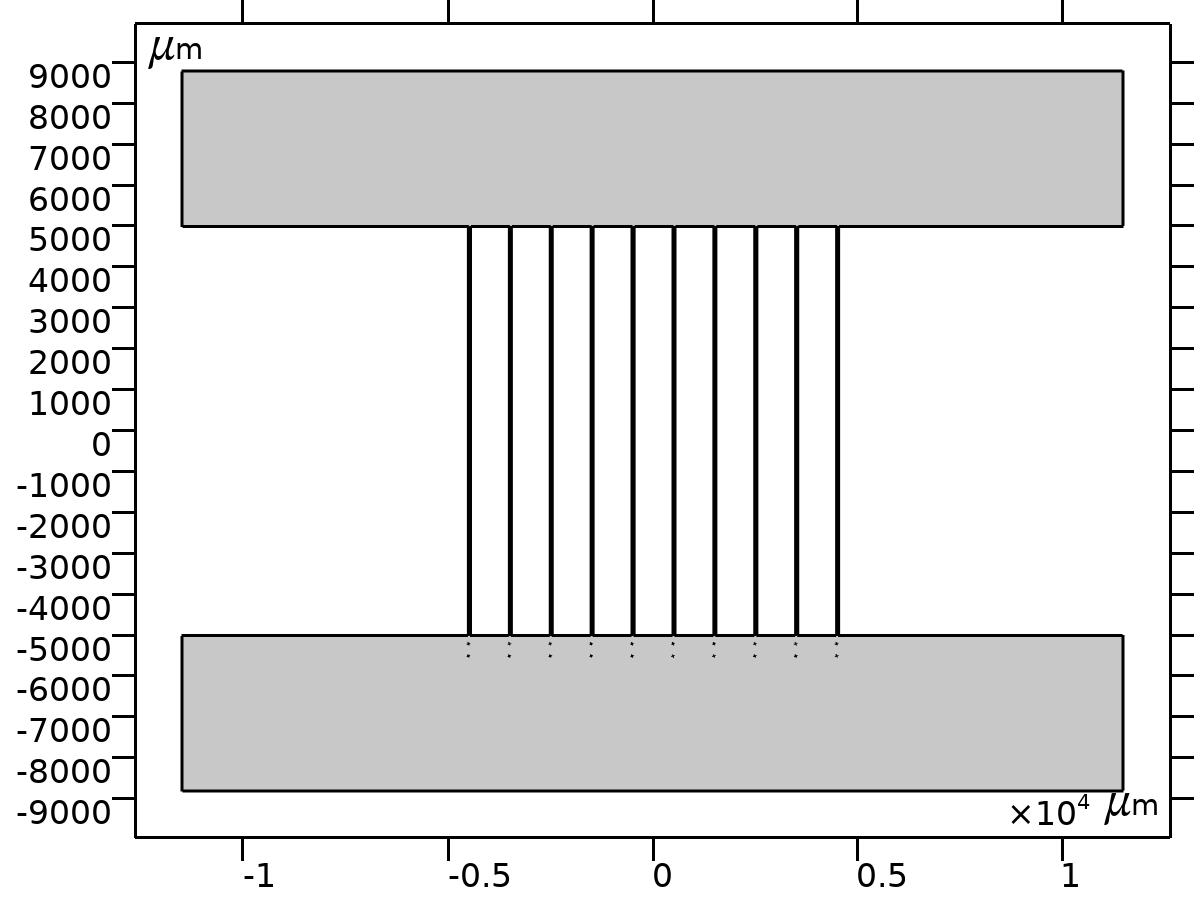


Units

| Length unit | µm |
| --- | --- |
| Angular unit | Deg |

- 1. Modeling Parameters

| Parameter | Value | Description |
| --- | --- | --- |
| ρ | 998.2 kg/m^3^ | Density of water |
| µ | 1.003e-3 Pa*s | Dynamic viscosity of water |

- 1. Laminar Flow


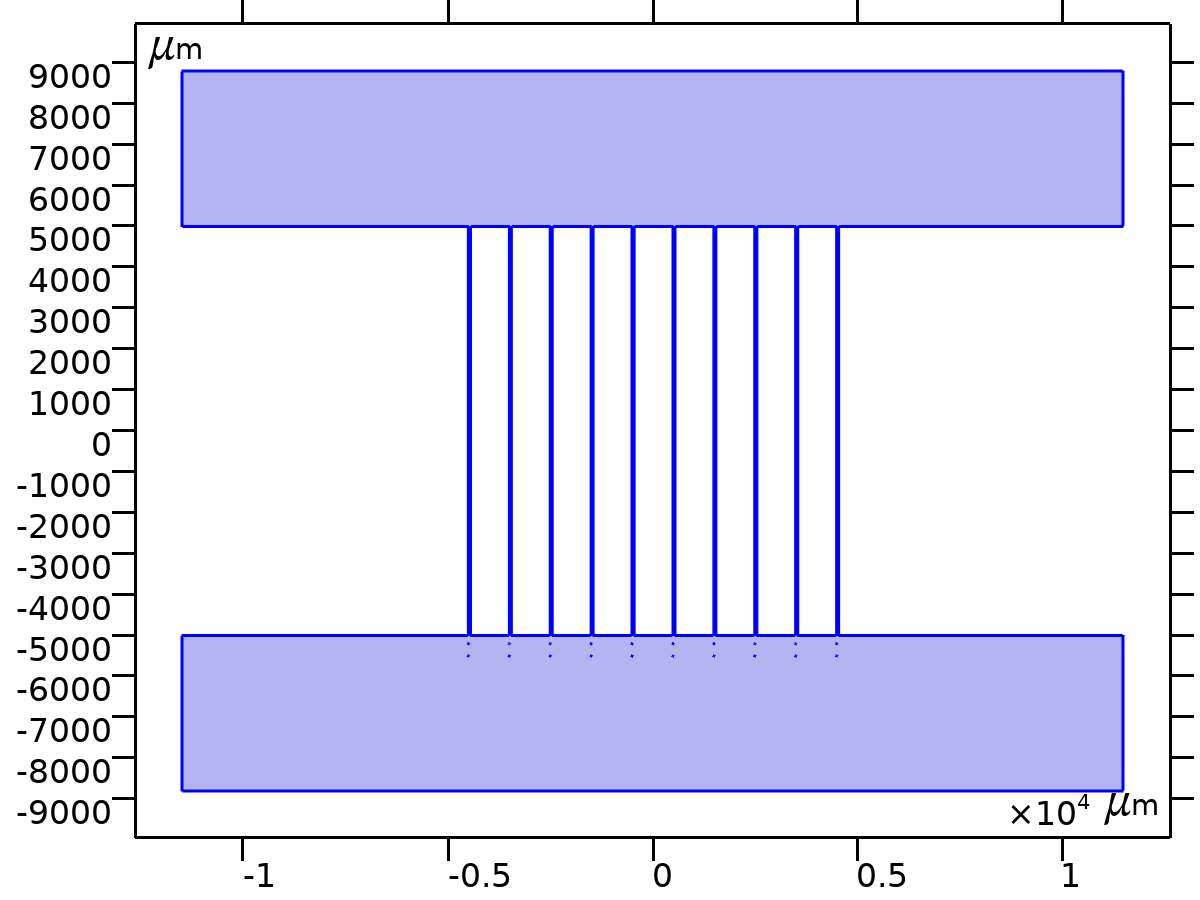


Equations


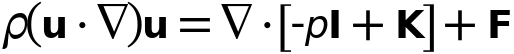


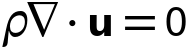


- - 1. Fluid Properties 1

Equations


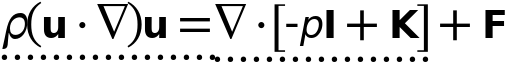


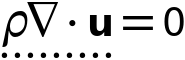


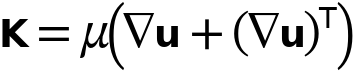


- - 1. Wall 1

Equations


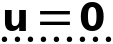


Boundary condition

Wall condition: No slip

- - 1. Inlet 1

Equations


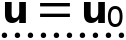


Boundary condition

Velocity

- - 1. Outlet 2

Equations


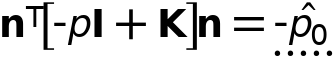


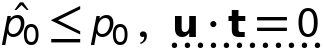


- - 1. Outlet 3

Equations


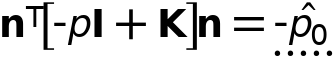


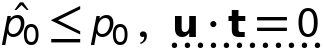


- - 1. Outlet 4

Equations


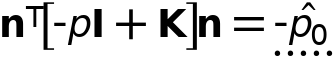


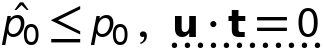

Supplement: Supplementary file 1 — Supplementary Information. [file 41598_2021_91117_MOESM1_ESM.docx]
